# Evaluation of the Effects of the Strengthening Families Program in Quebec Adolescents and Parents Living in Challenging Family Conditions

**DOI:** 10.1177/01632787251341460

**Published:** 2025-05-22

**Authors:** Sylvie Hamel, Carl Lacharité, Michael Cantinotti, Andrée-Anne Lepage, Jean Montambeault, Chantal Chicoine

**Affiliations:** 1 Department of Psychoeducation and Social Work, 14847 Université du Québec à Trois-Rivières, Trois-Rivières, QC, Canada; 2 Department of Psychology, 14847 Université du Québec à Trois-Rivières, Trois-Rivières, QC, Canada; 3 Équijustice Trois-Rivières, Trois-Rivières, QC, Canada

**Keywords:** strengthening families program, program evaluation, challenging family conditions, parent-child relation, adolescents’ social behaviour

## Abstract

The Strengthening Families Program (SFP) has recognized value in preventing criminality and drug use in adolescents from underprivileged backgrounds. The objective of this study was to measure the effects of a 14-week version of this program targeting adolescents 12–16 years old in two medium-sized Quebec municipalities, using a repeated-measure design. The study participants were: 1) an intervention group, consisting of 71 adolescents and 61 parents who participated in the SFP between January 2018 and December 2019; and 2) a comparison group, consisting of 57 adolescents and 56 parents. Multivariate longitudinal regression indicates that the SFP reinforces some protective factors, such as the parent-child relation, as well as some key dimensions of family strengths. However, no effect was observed on parenting practices or adolescents’ social behaviour. The differences between the intervention and control groups, the clinical significance of the results, and challenges of evaluating the SFP are discussed.

## Strengthening Families Program (SFP)

The SFP incorporates many characteristics of the best prevention programs targeting drug use, violence, and criminality by adolescents from underprivileged backgrounds (Midford, 2010; Miller & Hendrie, 2009). It’s based on a model of biopsychosocial vulnerability (Kumpfer & Magalhães, 2018). This ecological model posits that the development of certain high-risk behaviours, such as the consumption of psychotropic substances by adolescents is influenced by the environmental context (community, school, entourage, family). On the other hand, reinforcement of family ties, as well as cohesion, parental communication and supervision, positive family values, and access to, and recourse to, community resources are essential protective factors (Riesch et al., 2012). Positive parent-child relations, parental supervision, consistent parental discipline, and parental disapproval of drug use are among the main reasons adolescents avoid use of psychoactive substances and do not develop behavioural problems (Van Ryzin & Dishion, 2012). These factors not only favour children’s socialization, but also enhance families’ capacity to cope with difficulties as a unit (Kumpfer et al., 2003; Kumpfer & Magalhães, 2018).

Several versions of the SFP have been developed, for durations of 7–14 weeks and children of various age groups (and their families). The SFP family-based approach calls for every young participant to be accompanied by at least one parent, or an adult who plays a significant role in their life. The program covers three major themes, presented in the following order: family relations and attachment (importance of spending time together), communication (which should be clear and respectful), and limits (setting and respecting them).

## Research Objective and Hypothesis

The objective of this study was to measure the effects of a long version of the SFP (14 weeks) intended for adolescents 12–16 years old in families facing difficulties (Kumpfer & Magalhães, 2018). This program was delivered in two medium-sized municipalities in Quebec, with difficult living conditions, from January 2018 to December 2019. A total of 145 individuals (68 parents and 77 adolescents from 57 families) completed the SFP during this period. The main research hypothesis was that adolescents and parents in the intervention and comparison groups would develop differently^1^ over time in terms of: 1) the quality of the parent-child relationship (affection, openness, self-disclosure, support); 2) parenting practices (positive parenting practices, deficient parental supervision, inconsistent discipline); 3) family strengths (patterns of positive interaction, capacity to remain true to values, coping strategies of family members, commitment to, and confidence in each other, capacity to mobilize resources) and 4) adolescents’ social behaviour (rule breaking behavior, social problems).

## Method

### Study Design

This study adopted a quasi-experimental repeated-measurement design with a comparison group. T_0_, the first measurement time, corresponded to the beginning of the program. Participants were eligible if they had not attended more than three sessions of the program. T_1_ corresponded to one month after program completion (five months after T_0_) and T_2_ to six months after T_1_. T_3_ was added after the study had begun and corresponded to 5–37 months after T_2_^2^. These measurement times were used for both intervention and comparison groups.

### Recruitment

**Figure 1:**
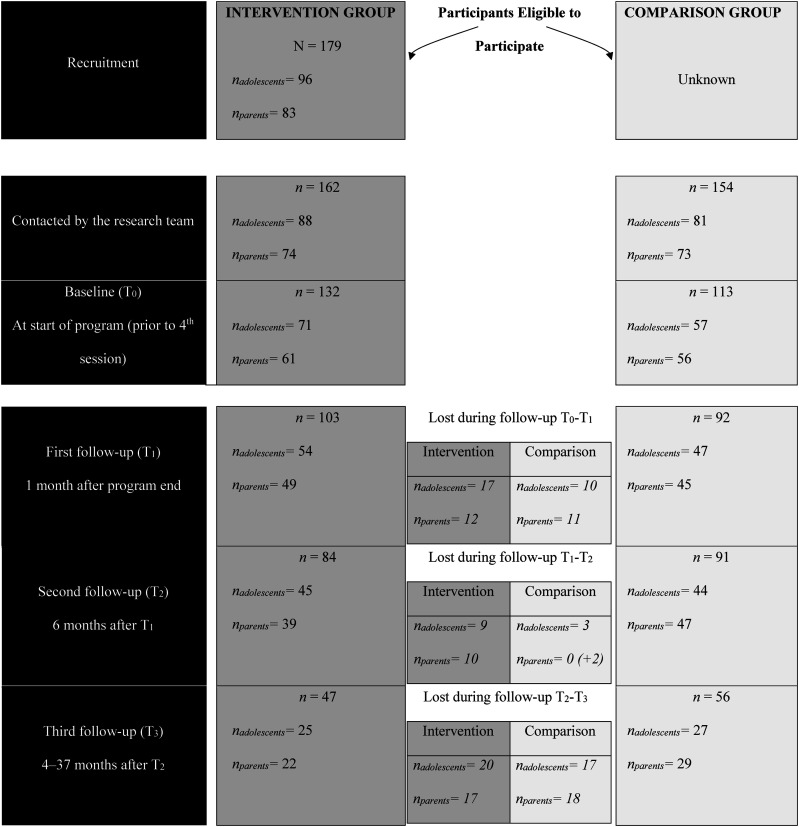
Flow Diagram on the Development of the Quasi-Experimental Study

The families who participated in the program were, at the point of departure, approached by institutions^3^ (64%), by community partners (33%) and parents having already participated in the SFP (3%). The recruitment of these families for research was then done by the SFP supervisors. In return, the recruitment for research of participants in the comparison group was done primarily by community workers (80%), then by institutional workers (20%). The number of participants (adolescents and parents) at each stage of the study, from recruitment for research to the last measurement period, is presented in Figure 1.

### Instruments

Four instruments were used to verify the study hypotheses in both adolescents and parents: 1) Claes et al.’s (2010) emotional availability and communication scales, to evaluate affection, openness, self-disclosure and support*;* 2) three scales from the French translation (Zlomke et al., 2014) of the *Alabama Parenting Questionnaire* (Shelton et al., 1996), to measure positive parenting practices, deficient parental supervision and inconsistent discipline; 3) the five scales from Deal et al.’s (1988) *Family Functioning Style Scale* that were adapted and translated into French to measure patterns of positive interaction, capacity to remain true to values, coping strategies of family members, commitment to, and confidence in each other and capacity to mobilize resources; and 4) two scales from a French translation (Vermeersch & Fombonne, 1997) of Achenbach’s (1991) *Child Behavior Checklist*, to measure social behaviour (rule-breaking behavior and perception of social problems). In all cases, we used a French version of these instruments.

### Procedure

The questionnaires were administered to each participant by research assistants. Prior to March 2020, participants were met at their homes or on the premises of community organizations. From March 2020 on, contact was by phone, due to COVID-19 restrictions. At the end of each questionnaire session, participants received a $20 compensation. This study was approved by the Research-Ethics Committees of the Université du Québec à Trois-Rivières and the CIUSSS-MCQ.

### Participant Profile

The average age of parents in both groups is 42 years old. The two groups also include an equal proportion of females (76%) and male (24%). Both groups also have similar profiles in education and employment. The average age of adolescents in both groups is between 13 and 14 years old. The two groups also include an equal number of girls (between 49% and 53%) and boys (between 47% and 51%) as well as a majority of youth born in the Quebec Province (over 90%). The adolescents in the intervention group had fewer years of education (17.9% compared to 9.4%), while those in the comparison group had slightly worse educational results (14.5% with yields below 60%, compared to 5.2%). Finally, the adolescents in the intervention group were more likely to be in a single-parent family and to have experienced (45.8% compared to 31.6 %), at some point in their lives, placement in a foster home or a care facility (5.2% compared to 1.8%).

In addition, attrition analyses were conducted, comparing the T_0_ scores of participants who had dropped out of the study at T_1_, T_2_, and T_3_ to the T_0_ scores of participants still present at those times. These confirm that both the adolescents and the parents in the intervention group had more issues at the T_0_ than their counterparts in the comparison group. In adolescents, this applies to the following variables: openness, self-disclosure and support. In parents, this applies to the following variables: *affection, openness, self-disclosure, support, inconsistent discipline, positive parenting practices, rule breaking behavior,* and *perceived social problems experienced by their child*.

### Data Analysis

The progress of the intervention and comparison groups (parents and adolescents analyzed separately) over time was analyzed through multivariate longitudinal regression with REML estimation, using Jamovi (v. 2.3.13) (Love et al., 2022), including the GAMLj module (v. 2.6.6) (Gallucci, 2025). This modelling technique accommodates measurement times that vary from participant to participant and missing values (Twisk, 2019). A binary COVID variable was included in the model to test whether participants who completed the questionnaires prior to March 13, 2020 (the date on which pandemic measures were introduced in Quebec) obtained scores similar to participants who completed the questionnaires after that date.

## Results

All the variables in the multivariate model are presented in Table 1 Among the notable results, the first involved *self-disclosure*, for which adolescents and parents in the comparison scored more poorly over time, adolescents of the intervention group exhibited stable scores, and parents of the intervention group scored somewhat better over time. For *capacity to remain true to values*, the comparison group (adolescents and parents) scored more poorly over time, while the intervention group (adolescents and parents) exhibited stable scores.

**Table 1:** Multivariate Longitudinal Regression Analyses

Variables	Intervention Group *M* [95% CI]			Comparison Group *M* [95% CI]			*F*	*p*
	25^e^	50^e^	75^e^	25^e^	50^e^	75^e^		
Affection A	3.28 [3.11, 3.44]	3.25 [3.10, 3.40]	3.22 [3.08, 3.36]	3.37 [3.22, 3.53]	3.32 [3.18, 3.46]	3.25 [3.10, 3.40]	1.78	.183
Affection P	3.16 [3.01, 3.31]	3.16 [3.02, 3.29]	3.15 [3.02, 3.28]	3.39 [3.26, 3.53]	3.37 [3.24, 3.50]	3.33 [3.20, 3.47]	1.20	0.275
Openness A	3.05 [2.88, 3.21]	3.04 [2.89, 3.19]	3.03 [2.89, 3.17]	3.18 [3.03, 3.33]	3.14 [3.00, 3.28]	3.09 [2.94, 3.23]	2.31	0.130
Openness P	3.25 [3.11, 3.38]	3.24 [3.12, 3.36]	3.23 [3.12, 3.34]	3.46 [3.35, 3.58]	3.44 [3.33, 3.55]	3.40 [3.29, 3.52]	1.54	0.216
Self-disclosure A**	2.38 [2.21, 2.56]	2.37 [2.21, 2.53]	2.36 [2.22, 2.50]	2.47 [2.31, 2.63]	2.40 [2.26, 2.55]	2.32 [2.16, 2.47]	5.27	0.022
Self-disclosure P***	2.54 [2.36, 2.71]	2.55 [2.38, 2.71]	2.56 [2.41, 2.71]	2.71 [2.55, 2.87]	2.65 [2.50, 2.81]	2.58 [2.43, 2.74]	9.43	0.002
Support A	3.10 [2.93, 3.28]	3.09 [2.93, 3.24]	3.07 [2.93, 3.21]	3.24 [3.09, 3.40]	3.21 [3.06, 3.35]	3.16 [3.01, 3.31]	0.71	0.401
Support P*	3.21 [3.06, 3.35]	3.20 [3.07, 3.34]	3.20 [3.08, 3.33]	3.40 [3.27, 3.53]	3.37 [3.24, 3.49]	3.32 [3.19, 3.45]	3.55	0.061
Inconsistent discipline A	13.7 [12.5, 14.9]	13.8 [12.7, 14.9]	13.9 [12.9, 14.8]	14.3 [13.3, 15.4]	14.5 [13.5, 15.5]	14.7 [13.7, 15.7]	0.25	0.621
Inconsistent discipline P	14.9 [13.7, 16.1]	14.8 [13.7, 15.8]	14.6 [13.6, 15.6]	13.7 [12.6, 14.7]	13.8 [12.8, 14.7]	13.9 [12.9, 14.9]	2.55	0.112
Positive parenting practices A	21.7 [20.4, 22.9]	21.7 [20.5, 22.8]	21.7 [20.6, 22.7]	22.2 [21.1, 23.3]	22.1 [21.0, 23.1]	21.9 [20.8, 22.9]	0.98	0.324
Positive parenting practices P	23.1 [22.1, 24.1]	23.1 [22.2, 24.0]	23.1 [22.2, 23.9]	24.2 [24.4, 25.1]	24.1 [23.3, 25.0]	24.0 [23.1, 24.9]	0.89	0.346
Deficient parental supervision A	22.1 [20.1, 24.0]	22.4 [20.6, 24.2]	22.8 [21.1, 24.5]	23.1 [21.3, 24.9]	23.2 [21.4, 24.9]	23.3 [21.5, 25.1]	0.77	0.381
Deficient parental supervision P	17.5 [15.8, 19.3]	18.3 [16.7, 19.8]	19.2 [17.8, 20.6]	17,8 [16.3, 19.3]	18.4 [17.0, 19.8]	19.1 [17.7, 20.6]	0.46	0.499
Commitment to, and confidence in each other A*	11.2 [10.5, 11.9]	11.2 [10.6, 11.8]	11.2 [10.6, 11.8]	11.7 [11.1, 12.3]	11.5 [11.0, 12.1]	11.3 [10.7, 11.9]	3.41	0.066
Commitment to, and confidence in each other P	13.1 [12.6, 13.5]	13.0 [12.6, 13.4]	12.9 [12.6, 13.3]	13.2 [12.8, 13.5]	13.1 [12.7, 13.4]	13.0 [12.6, 13.4]	0.04	0.844
Patterns of positive interaction A	34.7 [32.6, 36.8]	34.7 [32.7, 36.6]	34.6 [32.8, 36.4]	36.4 [34.5, 38.4]	36.0 [34.2, 37.9]	35.5 [33.6, 37.4]	1.89	0.170
Patterns of positive interaction P*	36.9 [35.2, 38.6]	37.1 [35.5, 38.7]	37.4 [35.9, 38.8]	39.0 [37.5, 40.6]	38.8 [37.4, 40.3]	38.5 [37.0, 40.0]	3.81	0.052
Capacity to mobilize resources A	6.75 [6.32, 7.17]	6.68 [6.31, 7.04]	6.58 [6.27, 6.90]	6.96 [6.60, 7.32]	6.92 [6.60, 7.23]	6.86 [6.53, 7.20]	0.15	0.704
Capacity to mobilize resources P	6.99 [6.44, 7.54]	6.96 [6.47, 7.45]	6.92 [6.47, 7.37]	7.09 [6.61, 7.56]	7.06 [6.62, 7.51]	7.03 [6.57, 7.49]	<0.01	0.944
Coping strategies of family members A*	13.0 [12.1, 13.8]	13.1 [12.3, 13.9]	13.3 [12.6, 14.0]	14.4 [13.6, 15.1]	14.3 [13.6, 15.0]	14.2 [13.4, 14.9]	2.96	0.087
Coping strategies of family members P	14.3 [13.6, 15.0]	14.3 [13.7, 15.0]	14.4 [13.8, 15.0]	14.6 [14.0, 15.2]	14.6 [14.0, 15.2]	14.6 [14.0, 15.2]	0.24	0.623
Capacity to remain true to values A**	18.3 [17.2, 19.3]	18.3 [17.4, 19.3]	18.4 [17.6, 19.3]	19.2 [18.3, 20.2]	19.0 [18.2, 19.9]	18.8 [17.9, 19.7]	4.50	0.035
Capacity to remain true to values P**	21.1 [20.3, 21.9]	21.1 [20.4, 21.8]	21.1 [20.4, 21.7]	22.4 [21.7, 23.0]	22.1 [21.5, 22.7]	21.7 [21.1, 22.4]	6.39	0.012
Rule breaking behavior A***	4.89 [3.69, 6.08]	5.17 [4.06, 6.29]	5.54 [4.49, 6.59]	5.56 [4.43, 6.69]	5.48 [4.38, 6.57]	5.37 [4.25, 6.48]	7.94	0.005
Rule breaking behavior P	7.26 [5.84, 8.69]	7.21 [5.88, 8.54]	7.14 [5.88, 8.40]	5.07 [3.75, 6.39]	5.05 [3.77, 6.33]	5.03 [3.72, 6.33]	0.05	0.816
Social problems A*	4.17 [3.30, 5.04]	4.29 [3.52, 5.06]	4.45 [3.76, 5.15]	5.23 [4.47, 5.99]	5.13 [4.42, 5.83]	4.99 [4.26, 5.72]	3.15	0.077
Social problems P	6.36 [5.25, 7.46]	6.17 [5.16, 7.18]	5.91 [4.97, 6.86]	4.82 [3.83, 5.81]	4.68 [3.73, 5.63]	4.50 [3.53, 5.47]	0.19	0.663

*Note.* A = adolescent, P = parent. For the regression coefficients, ‘group’ refers to the effect in the intervention group versus the effect in the comparison group, ‘time’ refers to the effect of a 1-day interval on the criterion variable (which explains why the coefficients appear low), ‘COVID’ is a covariable which indicates whether a participant was evaluated following the introduction of pandemic measures, and ‘group X time’ is an interaction effect.

*p < .10; **p < .05; ***p < .01 **** It should be noted that, unlike all other variables, the scores of the following four must be interpreted as a deterioration in the situation when the scores are higher: 1) Inconsistent discipline, 2) Deficient parental supervision, 3) Rule breaking behavior and 4) Social problems.

Differences were also observed for the two groups of parents: for both *support* and *patterns of positive interaction*, the parents in the comparison group exhibited decreasing scores over time while the parents in the intervention group exhibited stable or increasing scores.

Similarly, differences were observed in the two groups of adolescents. Thus, for *commitment to, and confidence in each other*, the scores of the comparison group decreased over time while those of the intervention group remained stable. For *coping strategies of family members*, the adolescents in the intervention group scored better over time while those in the comparison group evidenced decreasing scores. For *rule breaking behavior* and *social problems*, the situation was reversed: here, it was the intervention group that exhibited higher scores over time and the comparison group that exhibit lower ones.

## Discussion

The improvement of scores that we found in this research, concerning parent-child relation (*self-disclosure, support*) and family strengths (*capacity to remain true to values, patterns of positive interaction, commitment to, and confidence in each other, coping strategies of family members*), or otherwise their stability over time that we observe in the intervention group illustrate both the positive and protective effects of the SFP. But these results only partially support the research hypotheses, as no inter-group difference was observed for parenting practices, and adolescents in the intervention group. Those scored more poorly regarding social behaviours. While this suggests a differential progression, it obviously is not in the direction expected.

Clearly, these results cannot be interpreted without also considering the differences in the two groups at the outset of the study (T_0_). While the adolescents of the intervention group scored better than the adolescents of the comparison group for *self-disclosure, commitment to, and confidence in each other, patterns of positive interaction*, *capacity to remain true to values* and *coping strategies of family members* in the multivariate analysis, they scored more poorly in the attrition analysis. This finding in some ways converges with the positive and sustainable—if not frankly protective—effects of the SFP observed among youth in terms of parent-child relation and family strengths. These effects are particularly striking considering that the parents in the intervention group scored more poorly at T_0_ than the parents in the comparison group, for *self-disclosure*, *support* and *capacity to remain true to values*. These robust findings demonstrate the SFP’s capacity to reinforce protective factors of families facing difficult situations.

However, it was also true that multivariate analysis revealed no effect of the SFP on parenting practices, in either adolescents or parents, and that the social behaviours (rule breaking behavior and social problems) of the adolescents in the intervention group deteriorated over time, compared to the adolescents in the comparison group. In this context, it should be recalled that comparative analyses at T_0_ revealed poorer scores for these variables for the parents in the intervention group. This mirrors the fact that 64% of SFP participants had been referred by case workers of the CIUSSS-MCQ, an institution that provides support for adolescents and families requiring front and second-line resources, and sometimes even interventions mandated by the Quebec Youth Protection Act (Gouvernement du Québec, 2022). As a result, the fact that the comparison groups were not completely comparable to the intervention groups hindered demonstration of SFP effects for these variables.

It is nevertheless important to not lose sight of the fact that the multivariate regression indicates a deterioration of the social behaviours of adolescents, but not of parents’ perception of these behaviours. Clearly, parents and adolescents view the latter’s social behaviours differently. We believe that one explanation may be that the adolescents matured over the study period and that the program strengthened their capacity to “open up” and become self-aware, ultimately helping them to become more self-conscious of their behaviours. It is also possible that parents may have found it difficult to report behaviours by their children who were approaching adulthood and attempting to become independent. In addition, the protective effect of parenting practices on the development of delinquency decreases as adolescents grow older (Jalling et al., 2016). Finally, empirical evidence from more than 30 years of research indicates that adolescent involvement in criminal activity generally intensifies in early adolescence, peaks at 16–17 years of age, and tends to decline towards the end of adolescence and in early adulthood (Farrington, 2005).

Moreover, it should be noted that other studies, such as those by Skärstrand et al. (2014) in Sweden, Baldus et al. (2016) in Germany, and Foxcroft et al. (2017) in Poland, also failed to detect the effects of the SFP on adolescents’ social behaviour, specifically drug use. Although these three studies evaluated the effects of a variant of the SFP targeting children 10–14 years old (rather than 12–16 years old, as in the current study), Gorman’s (2017) replication study is revealing. Gorman noted that psychological research often relies on variables and concepts with varying definitions and different research designs. The present study, like the three studies mentioned above, had no affiliation with the research group headed by the program’s originator, Karol Kumpfer. In the present study, tools in wide use in Quebec were used to understand the viewpoint of adolescents as well as parents. This approach is clearly different from the evaluation of the variant of the SFP intended for adolescents 12–16 years old by Kumpfer et al. (2012), who used a retrospective design, focused on the point of view of parents, and used standard scores as the basis of comparison.

While this study did not identify any effects of the SFP on parenting practices and adolescents’ social behaviours, its primary finding is that the SFP reinforces key elements of family strengths (capacity to remain true to values, patterns of positive interaction, commitment to, and confidence in each other and coping strategies of family members), and the parent-child relation (particularly self-disclosure). These results are consistent with Orte et al. (2019), who reported that the variant of the SFP targeting 12–16-year-olds reinforces the family resilience, by strengthening family affection and support, communication, and emotional resilience. Additionally, the parent-child relation, like parenting practices, is a recognized fundamental proximal preventive factor for adolescents’ behaviour problems. The scientific literature indicates that the relationship between adolescents adopting antisocial behavior and their parents is characterized by a lack of intimacy and reciprocity, and that negative parent-child relations are associated with higher levels of externalized problems (Deković et al., 2003).

Adolescence is a critical developmental stage, and requires adolescents to redefine their relationships with their parents (Smith, 2012). On the one hand, parents must recognize and adapt to the fact their adolescent child is no longer a mere child, and on the other, adolescents must set aside the benefits of childhood dependence. Consequently, adolescence modifies the parent-child relation and the global functioning of the family. It is in fact one step in the lifecycle of the family, in which the adolescent is a key player (Smith, 2012).

These observations shed a new light on the positive effects of the SFP observed in the present study. By targeting adolescents, the SFP directly addresses individuals at risk of delinquency and offers them the opportunity to assume a different, undoubtedly more positive, role in their family. The SFP’s global and systemic approach in fact encompasses all members of the family. The things the adolescents and their parents learn in the program, as well as the changes they make to their day-to-day relations, necessarily have ramifications for all members of the family.

### Limitations

An important limitation of this study was the lack of an equivalent comparison group, which clearly limited its ability to demonstrate some program effects. This is a common and well-known challenge in evaluative research and was encountered in other evaluative studies of the SFP reported in the literature. In these latter cases, it appears that the between-group differences were less numerous and less significant, and that complementary analyses were able to palliate this weakness. However, in the present study, it was difficult to overcome this limitation. Nevertheless, we did consider it important to highlight the between-group differences, as these are eloquent illustrations of the real-world challenges associated with the delivery of programs to vulnerable populations. The level of attrition that was observed in this study also limits the external validity of the results. Further studies might benefit from including additional follow-up strategies as those outlined by van der Ven et al. (2022) to limit lost to follow-up participants.

## Conclusion

On the one hand, the results of this study reveal that the variant of the SFP targeting 12–16-year-olds reinforces the parent-child relation as well as certain key determinants of family strengths. These results are particularly interesting in light of the fact that they are based on a variant of the program that has not been extensively evaluated and demonstrate that adolescents too can benefit from the SFP. In addition, the SFP’s global and systemic approach offers adolescents the opportunity to play a different role in their family, as a role model for all family members.

Furthermore, the results of this study highlight the challenges of program evaluation in general. These challenges have critical implications, as they can mask certain effects of programs and consequently result in decisions that are ill-founded or threaten the long-term prospects of a program. Program evaluation thus places a heavy burden on researchers, and methodological rigour is essential if the true effects of a program are to be identified. Transparency concerning challenges in study design and the impact of these challenges on the results obtained is undeniably even more important.

## Supplemental Material

Supplemental Material - Evaluation of the Effects of the Strengthening Families Program in Quebec Adolescents and Parents Living in Challenging Family Conditions Supplemental Material for Evaluation of the Effects of the Strengthening Families Program in Quebec Adolescents and Parents Living in Challenging Family Conditions by Sylvie Hamel, Carl Lacharité, Michael Cantinotti, Andrée-Anne Lepage, Jean Montambeault, and Chantal Chicoine in Evaluation & the Health Professions

## Notes

1 The formulation of our hypothesis corresponds to the bidirectional tests used to test for positive and negative effects, since the SFP had only been implemented in the two target municipalities recently, which could have resulted in paradoxical results that would have escaped detection with unidirectional tests.

2 For some participants, therefore, the T0–T3 interval was 4 years.

3 The institutional partner offers front- and second-line services to young people 0–21 years old with mental-health or dependency issues. It also is responsible for young people take into care or followed under the provisions of Quebec’s Loi sur la Protection de la jeunesse [youth protection act].
