# Supplementary material for: Evaluation of the Effects of the Strengthening Families Program in Quebec Adolescents and Parents Living in Challenging Family Conditions
Source: Eval Health Prof. 2025 May 22;49(1):92–9. doi: 10.1177/01632787251341460 (PMC12847457; doi:10.1177/01632787251341460)

Figures

This Supplemental Material section presents figures for 10 significant (p < .01) or almost significant (p < .05) results that were found for the principal effect of interest, *group X time*, with multivariate analyses.

Self-disclosure

Figure 1

Self-disclosure - adolescents
Group X time effect for comparison and intervention group of adolescents

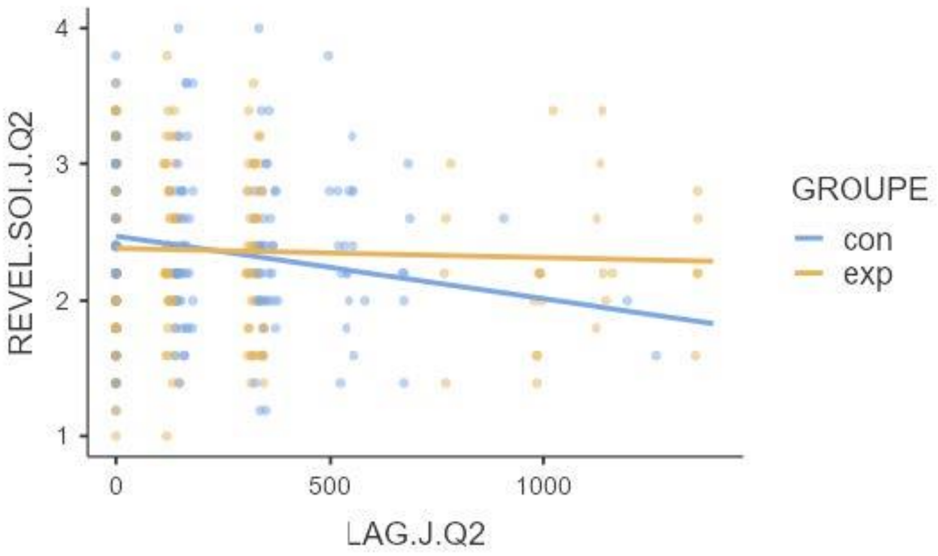

Figure 2

Self-disclosure - parents
Group X time effect for comparison and intervention group of parents

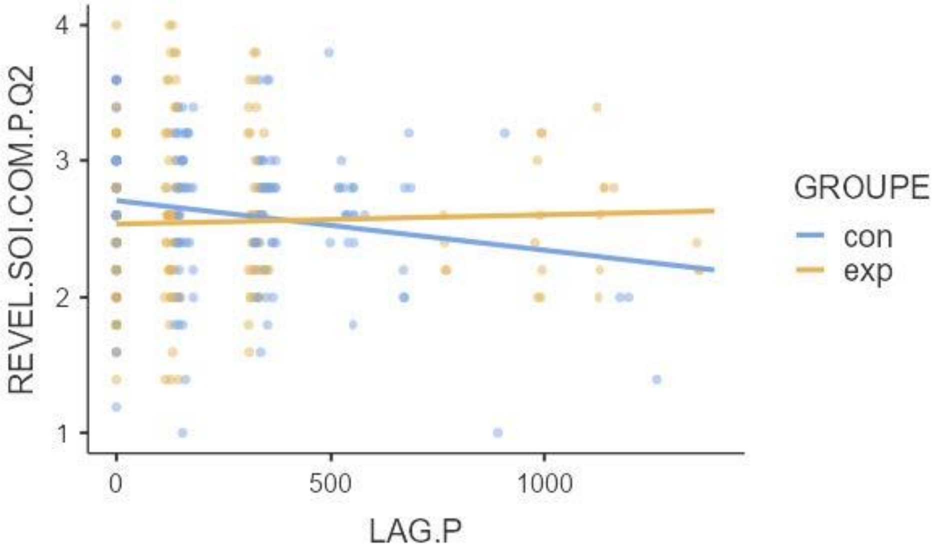

Coping Strategies of Family Members

Figure 3

Coping strategies of family members - adolescents
Group X time effect for comparison and intervention group of adolescents

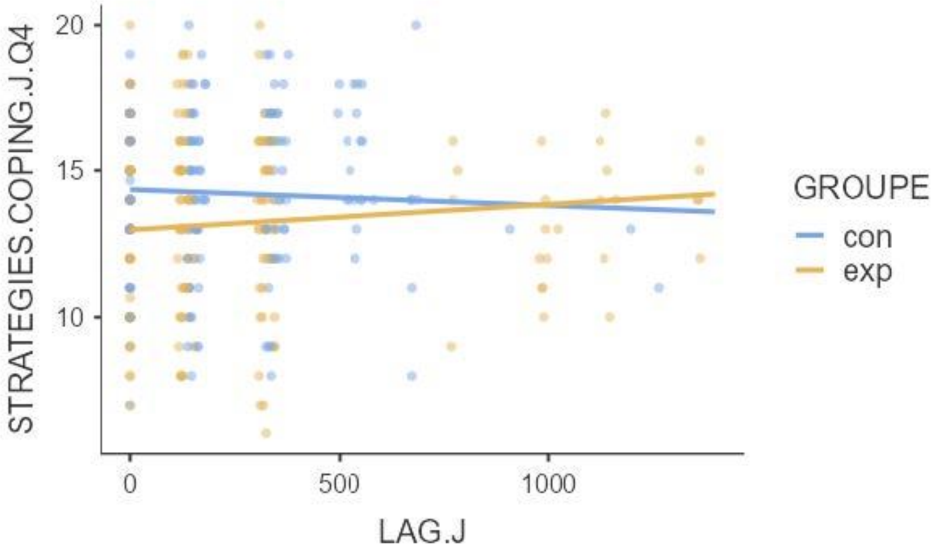

Commitment to, and Confidence in Each Other

Figure 4

Commitment to, and confidence in each other - adolescents
Group X time effect for comparison and intervention group of adolescents

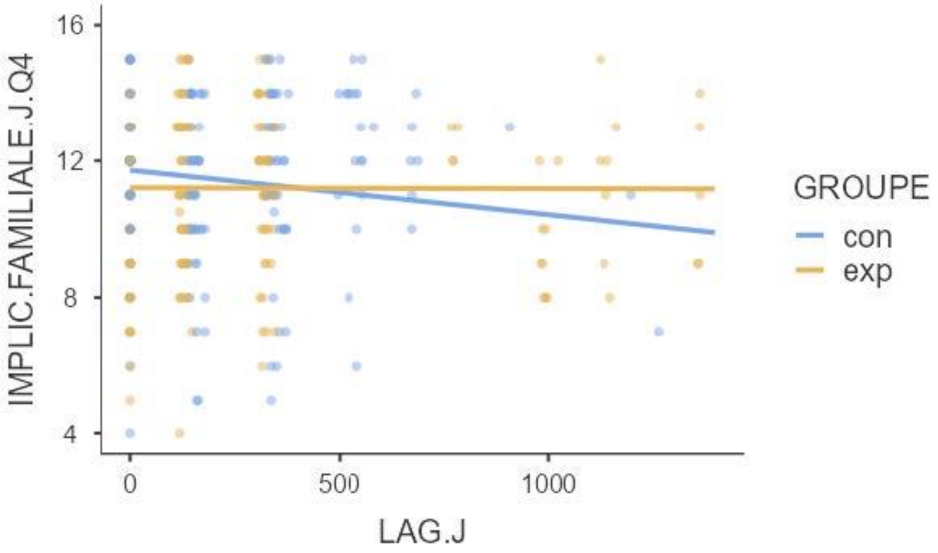

Rule Breaking Behavior

Figure 5

Rule breaking behavior - adolescents
Group X time effect for comparison and intervention group of adolescents

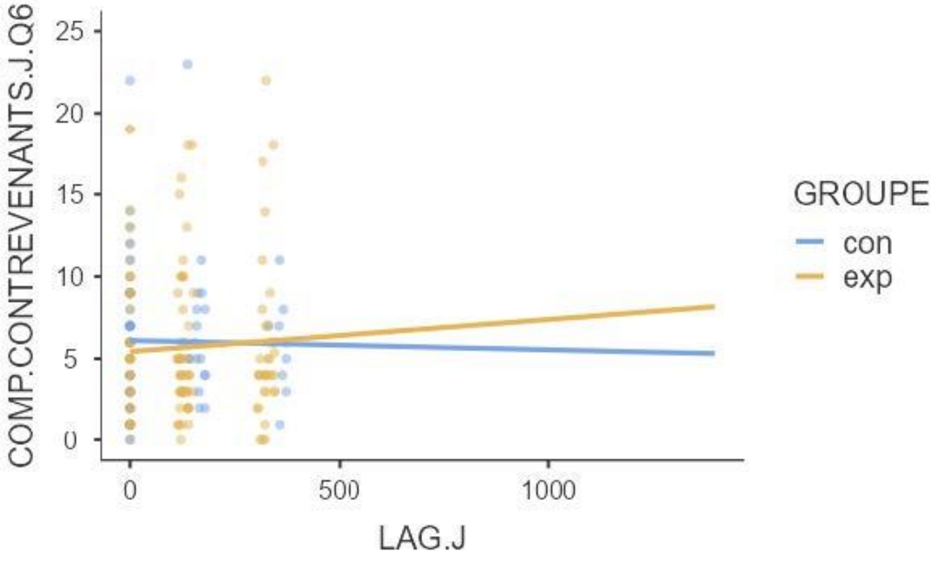

Social Problems

Figure 6

Social problems - adolescents
Group X time effect for comparison and intervention group of adolescents

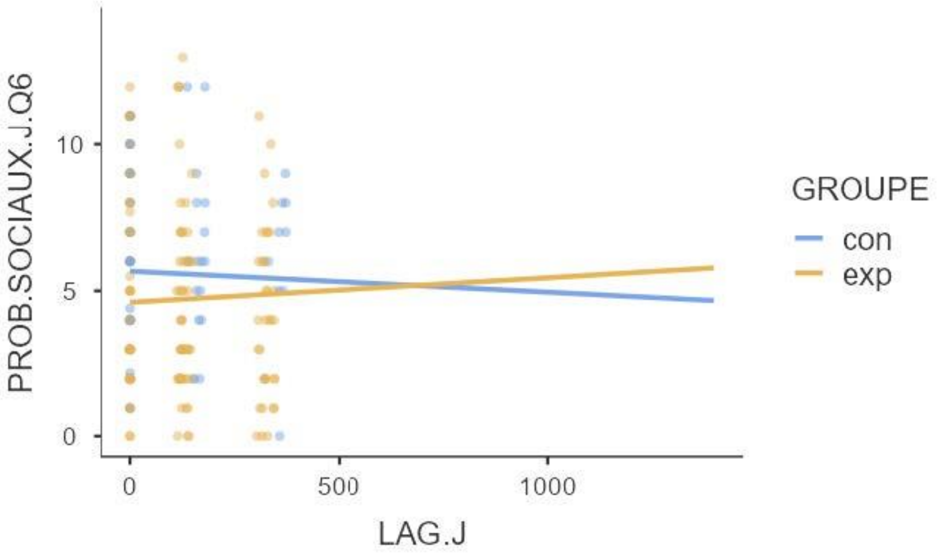

Support

Figure 7

Support - parents
Group X time effect for comparison and intervention group of parents

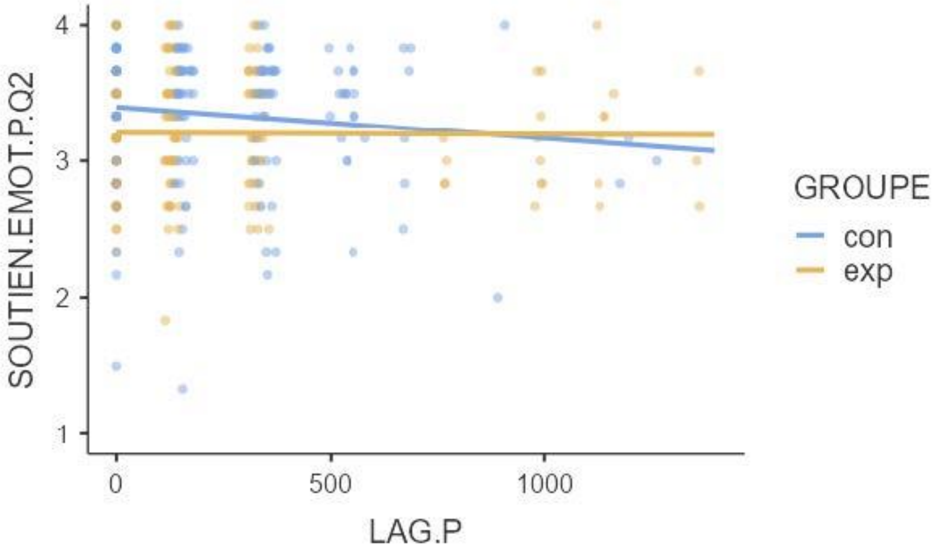

Patterns of Positive Interaction

Figure 8

Patterns of positive interaction - parents
Group X time effect for comparison and intervention group of parents

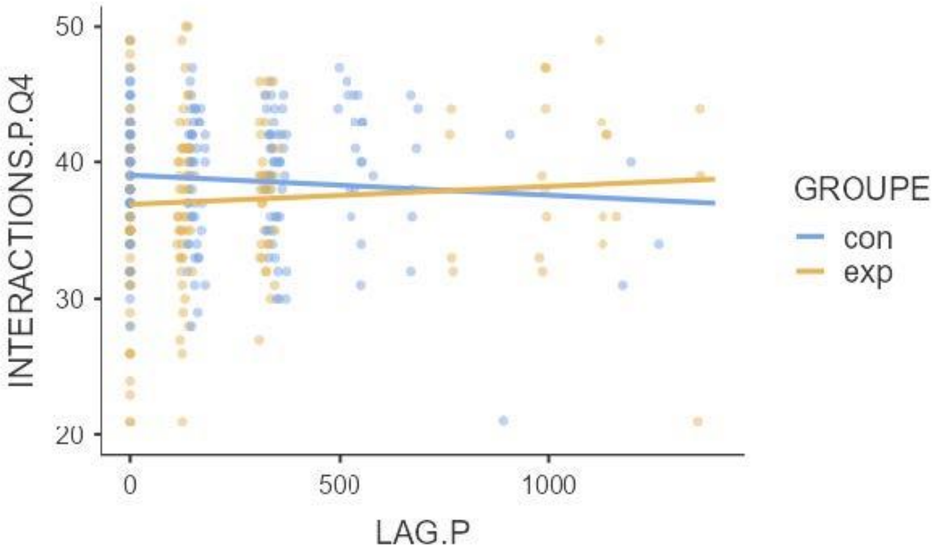

Capacity to Remain True to Values

Figure 9

Capacity to remain true to values - adolescents
Group X time effect for comparison and intervention group of adolescents

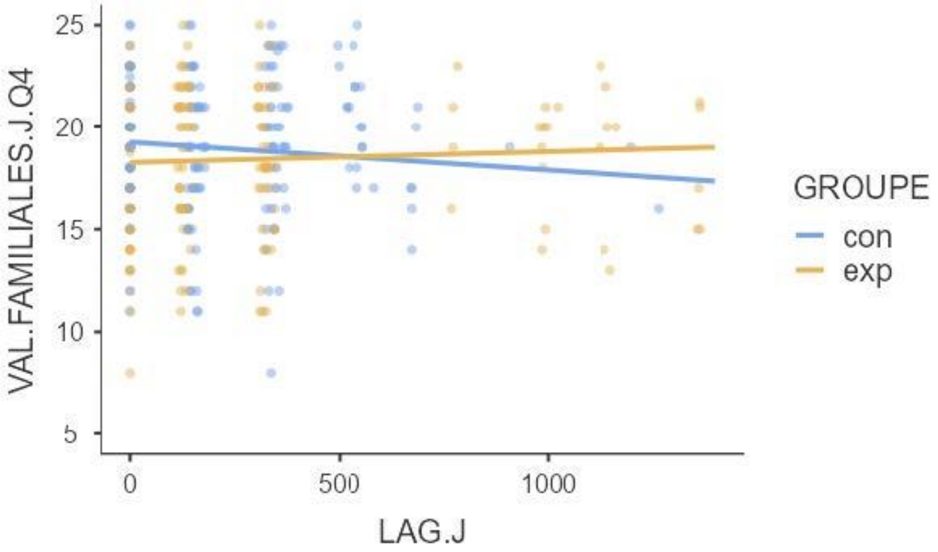

Figure 10

Capacity to remain true to values - parents
Group X time effect for comparison and intervention group of parents

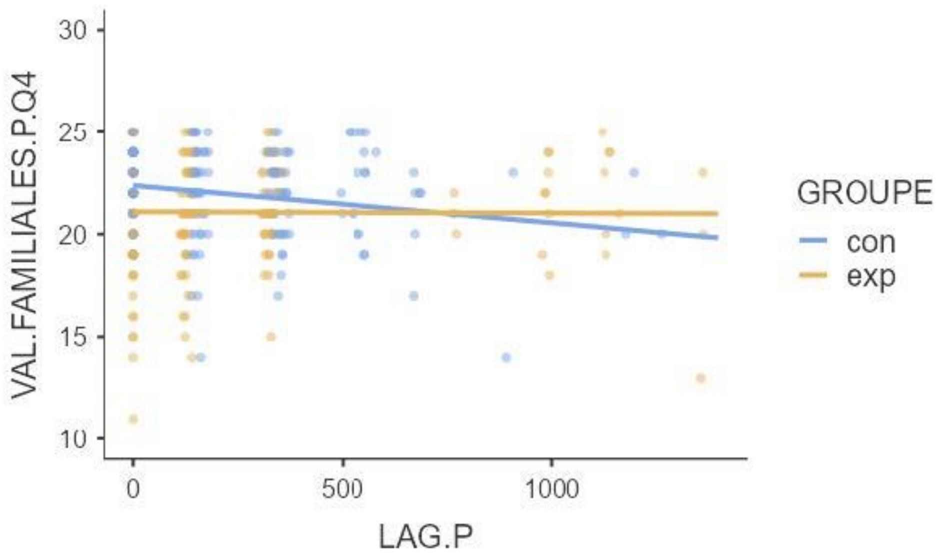

Supplement: Supplemental Material - Evaluation of the Effects of the Strengthening Families Program in Quebec Adolescents and Parents Living in Challenging Family Conditions [file sj-pdf-1-ehp-10.1177_01632787251341460.pdf]
